# Lyon-IARC Polyomavirus DNA in Feces of Diarrheic Cats

**DOI:** 10.1128/MRA.00550-19

**Published:** 2019-07-18

**Authors:** Elizabeth Fahsbender, Eda Altan, Marko Estrada, M. Alexis Seguin, Pauline Young, Christian M. Leutenegger, Eric Delwart

**Affiliations:** a Vitalant Research Institute, San Francisco, California, USA; b Department of Laboratory Medicine, University of California San Francisco, San Francisco, California, USA; c IDEXX Laboratories, Inc., West Sacramento, California, USA; Portland State University

## Abstract

A viral metagenomic analysis of feces from an unexplained outbreak of feline diarrhea revealed the presence of Lyon-IARC polyomavirus (LIPyV) DNA. LIPyV, whose genome was originally sequenced from swabs of human skin, was fecally shed by three out of five diarrheic cats.

## ANNOUNCEMENT

Stool samples were collected from five cats during a diarrhea outbreak in a hoarding situation in Guelph, Canada, and tested for Campylobacter coli, Campylobacter jejuni, Tritrichomonas blagburni (formerly Tritrichomonas foetus), Giardia spp., Cryptosporidium spp., Toxoplasma gondii, Salmonella spp., Clostridium perfringens alpha toxin (CPA) gene, Clostridium perfringens enterotoxin (CPE) gene, feline coronavirus, and feline panleukopenia virus (FPLV) using the IDEXX feline comprehensive diarrhea RealPCR panel. One sample was positive for both FPLV and Clostridium perfringens alpha toxin gene. FPLV, a protoparvovirus closely related to pathogenic canine parvovirus 2, can cause panleukopenia and has been associated with diarrhea but is also a common subclinical infection in cats (1, 2).

Stool samples from these 5 cats were pooled, supernatant filtered, and digested with a mixture of nuclease enzymes to reduce free nucleic acid content. Remaining capsid-protected nucleic acids were then extracted (3). Nucleic acids were incubated for 2 min with 100 pmol of primer A (5′-GTTTCCCACTGGANNNNNNNN-3′), followed by a reverse transcription step using Superscript III reverse transcriptase (Invitrogen) with a subsequent Klenow DNA polymerase step (New England Biolabs). cDNA was then amplified by a PCR step using AmpliTaq Gold DNA polymerase low-DNA (LD) with primer A-short (5′-GTTTCCCACTGGATA-3′).

Random reverse transcription-PCR (RT-PCR) was then followed by use of the Nextera XT DNA library preparation kit (Illumina, San Diego, CA) (3). Sequencing was performed on a MiSeq platform (250-bp paired-end reads), generating 1,216,275 reads that were *de novo* assembled using the Ensemble program (4). Both contigs and singlets were then analyzed using translated protein sequence similarity search (BLASTx) to all annotated viral proteins available in GenBank.

Two mammalian viruses were detected by BLASTx search (E score, <10^−30^), Lyon-IARC polyomavirus (LIPyV) (GenBank accession number NC_034253), an alphapolyomavirus in the family Polyomaviridae (5), and bocaparvovirus 3 (NC_039044) in the genus Bocavirus of the family Parvoviridae. Individual stool samples were then tested for these viruses using nested PCR. For LIPyV DNA, the first-round primers were LIPyV37F (5′-GCTTCATGAGGCTTCAAAGG-3′) and LIPyV338R (5′-TTGCATGTACCTTTTGCTCTG-3′), followed by the second-round primers, LIPyV_75F (5′-CATAATGCAGGGTCATTTGC3-3′) and LIPyV258R (5′-AAAGAGCCGTTTTGCCTTTT-3′). For bocavirus, the first-round primers were FBP54F (5′-AGCCATACAGTTTGACGGCA-3′) and FBP335R (5′-TGCTGCATGTTCGAATCACG-3′), and the second-round primers were FBP100F (5′-TTTGCCGGTACTAGCTGGAC-3′) and FBP307R (5′-CACCGCTTTGAGCTACTTGC3-′). Forty cycles of PCR each were used with 52°C and 54°C annealing temperature for the first and second rounds, respectively. PCR products of the correct size were identified by gel electrophoresis and confirmed by Sanger sequencing. Three fecal samples tested positive for LIPyV (one coinfected with FPLV), and a fourth was positive for bocaparvovirus 3 DNA. Bocaparvovirus 3 has been reported in feline feces and tissues, but its pathogenicity has not been established (6–8). The feline origin of the fecal samples was confirmed by PCR and Sanger sequencing of a 16S ribosomal DNA (rDNA) marker (9). One LIPyV DNA-positive sample was then selected for further deep sequencing using the Ovation Ultralow library system V2 kit (Nugen, Redwood City, CA), generating 1,452,148 Illumina reads, of which 109 belonged to LIPyV, covering 65.7% of the genome. PCR primers were designed to span two remaining gaps, and the amplicons were Sanger sequenced to complete the LIPyV genome. The 5,263-bp genome of the LIPyV (MK898813; GC content of 38%) showed 97% nucleotide similarity to the 5,269-bp reference LIPyV genome (NC_034253) using BLASTn. The next closest polyomavirus relative was isolated from raccoons, in which it induces brain tumors (10, 11), with LT and VP1 proteins showing 51 and 66% protein identity to those of LIPyV, respectively. Based on International Committee on Taxonomy (ICTV) LT-based classification (12), LIPyV is an alphapolyomavirus. An alternative taxonomic approach (13) would classify LIPyV in the Almi clade in both the LT and VP1 proteins.

The LIPyV genome was originally discovered using a degenerate PCR approach on human saliva samples and skin swabs (5). LIPyV-specific PCR detected polyomavirus DNA in 9/445 skin swabs and 1/439 plucked eyebrow hair samples from healthy U.S. adults and in 3/140 gargle samples from healthy French adults (5). LIPyV was also found by PCR in 1/127 benign skin samples from liver transplant recipients in Finland (14) but not in 689 noncancerous human tonsil samples or 139 gargle samples from France (15). The initial publication describing the LIPyV genome stated that “we cannot exclude the possibility that LIPyV is an animal virus and its presence in the human body may represent an environmental contamination” (5). A serosurvey of 152 human serum samples from the Netherlands did not detect any LIPyV seroreactivity, indicating either no or only rare human infections (16). Testing for anti-LIPyV antibodies and/or for viral RNA expression in cats will be required for a definitive host species assignment for LIPyV. The role of LIPyV in feline diarrhea, if any, with or without aggravating coinfections, remains to be determined.

### Data availability.

The complete genome sequence of this feline feces-associated LIPyV has been deposited in GenBank under the accession number MK898813. The raw data for both the Nugen and Nextera libraries are available under the accession number PRJNA541523.
